# Tracing the retina to analyze the integrity and phagocytic capacity of the retinal pigment epithelium

**DOI:** 10.1038/s41598-020-64131-z

**Published:** 2020-04-29

**Authors:** Francisco J. Valiente-Soriano, Manuel Salinas-Navarro, Johnny Di Pierdomenico, Diego García-Ayuso, Fernando Lucas-Ruiz, Isabel Pinilla, Nicolás Cuenca, Manuel Vidal-Sanz, María Paz Villegas-Pérez, Marta Agudo-Barriuso

**Affiliations:** 1 Departamento de Oftalmología, Facultad de Medicina, Universidad de Murcia and Instituto Murciano de Investigación Biosanitaria-Virgen de la Arrixaca (IMIB-Arrixaca), Murcia, Spain; 2 Instituto de Investigación Sanitaria Aragón, Aragon Health Sciences Institute, Lozano Blesa University Hospital Zaragoza, Zaragoza, Spain; 3 Departamento de Fisiología, Genética y Microbiología, Universidad de Alicante, Alicante, Spain

**Keywords:** Neuroscience, Retina

## Abstract

We have developed a new technique to study the integrity, morphology and functionality of the retinal neurons and the retinal pigment epithelium (RPE). Young and old control albino (Sprague-Dawley) and pigmented (Piebald Virol Glaxo) rats, and dystrophic albino (P23H-1) and pigmented (Royal College of Surgeons) rats received a single intravitreal injection of 3% Fluorogold (FG) and their retinas were analyzed from 5 minutes to 30 days later. Retinas were imaged *in vivo* with SD-OCT and *ex viv*o in flat-mounts and in cross-sections. Fifteen minutes and 24 hours after intravitreal administration of FG retinal neurons and the RPE, but no glial cells, were labeled with FG-filled vesicles. The tracer reached the RPE 15 minutes after FG administration, and this labeling remained up to 30 days. Tracing for 15 minutes or 24 hours did not cause oxidative stress. Intraretinal tracing delineated the pathological retinal remodelling occurring in the dystrophic strains. The RPE of the P23H-1 strain was highly altered in aged animals, while the RPE of the RCS strain, which is unable to phagocytose, did not accumulate the tracer even at young ages when the retinal neural circuit is still preserved. In both dystrophic strains, the RPE cells were pleomorphic and polymegathic.

## Introduction

The retinal pigment epithelium (RPE) is a monolayer of pigmented cells located between the neurosensory retina and the choroid. The RPE forms the outer blood retinal barrier and performs several functions essential for the retina, such as active transport of ions and other substances, light absorption, photopigment renewal, trophic factor secretion, immune modulation and phagocytosis of the photoreceptor outer segment membranes^1–4^. Thus, the RPE is indispensable for visual function and photoreceptor survival.

RPE cells are hexagonal and in the human retina their size and density changes depending on their eccentricity, being larger, flatter and less pigmented in the peripheral retina than in the macula^5^. The RPE contains two types of cellular pigments, melanin and lipofuscin. Melanin is a black pigment stored in melanosomes. Lipofuscin forms yellow-brown autofluorescent granules that increase with age and are believed to be the result of undigested phagosomes^1,6^.

Several studies have described age-related structural, morphological and functional changes of the human RPE^1,7–12^. There are also RPE alterations in several human retinal pathologies^9,13–17^ such as retinitis pigmentosa (RP) or age-related macular degeneration (AMD). RP is the most frequent form of inherited photoreceptor degeneration, while AMD is a multifactor disease modulated by genetic and environmental components^9,13,14^. It is believed that in the aged, RPE may be dysfunctional due oxidative stress and inflammation and this may contribute to AMD pathology^2,9,18^. Moreover, in some forms of RP, RPE phagocytosis is impeded. Furthermore, in the latest stages of this disease RPE cells usually migrate, invade the retina and participate in the process of retinal remodelling^18–24^.

The RPE is studied *in vivo* with optical coherence tomography (OCT) and fundus autofluorescence (FAF), and *ex vivo* using immunodetection. OCT allows the imaging of the retinal layers and their morphological changes in different pathologies, both in humans and animal models^8,16,25–31^. The metabolic state and the integrity of the RPE is studied with FAF to visualize the fluorescence emitted by the lipofuscin present in the RPE cells^32–38^. Lipofuscin accumulations are an indicator of the RPE malfunction or atrophy that is observed in multiple retinal pathologies^39,40^. In addition, FAF is used in ophthalmological practice for the study and assessment of the different patterns of drusen, pigmentary changes, geographical atrophies or neovascular alterations^33,40–42^.

*Ex vivo*, the RPE can be identified and studied using immunodetection of specific proteins, such as the tight junction protein 1 (zonula occludens 1, ZO-1) or the retinal pigment epithelium-specific 65 kDa (RPE65) protein. The most commonly used is ZO-1, a major structural protein of intercellular junctions that regulates RPE proliferation^43–46^. Thus, ZO-1 immunodetection allows the precise study of the morphology and density of RPE cells and provides a similar staining to cadherin, that is expressed in the zonula adherens junction of epithelial cells^47,48^.

Here we describe a new approach to label the retinal neurons and the RPE and to assess the integrity and phagocytic capacity of the RPE. This technique is based on the tracing of the retina after intravitreal administration of fluorogold. Fluorogold (FG^49^) is a fluorescent retrograde axonal tracer that is taken up by the axonal terminals or non-myelinated axons and then transported to the neuronal somas. FG accumulates in vesicles mainly in the cytoplasm without staining the nucleus, although the cytoplasmic membrane and nucleolus may take up some of the tracer. FG tracing is long-lasting and so the neurons remain labeled for up to three weeks^50–53^. As many other tracers, FG can undergo bidirectional transport within the neuron, although the retrograde transport prevails^51,54,55^.

To confirm the validity of this technique we have analysed and compared young and old retinas from control healthy rat strains (Sprague Dawley and Piebald Virol Glaxo), and two dystrophic rat strains: P23H-1 and RCS. P23H-1 rats suffer a mutation in the rhodopsin gene that causes first the death of rods and later of cones^19,56–58^. The RCS rats have a mutation of the *MERKT* gene, a tyrosinase kinase receptor necessary for RPE phagocytosis^21,23,24,56,58–61^. In this strain, the RPE is defective for phagocytosis causing therefore the degeneration of rods and cones.

## Results

### Time course of intraretinal tracing by intravitreal administration of fluorogold

We administered fluorogold intravitreally to establish a new method to trace retinal cells. In pilot experiments (Supplementary Data 1) from 1.5 to 5 µl of 3% FG were injected into the vitreous. The best tracing was obtained with 1.5 µl, since with higher volumes some FG remained in the vitreous impeding retinal visualization. Thus, all the experiments were done with this volume.

**Figure 1:**
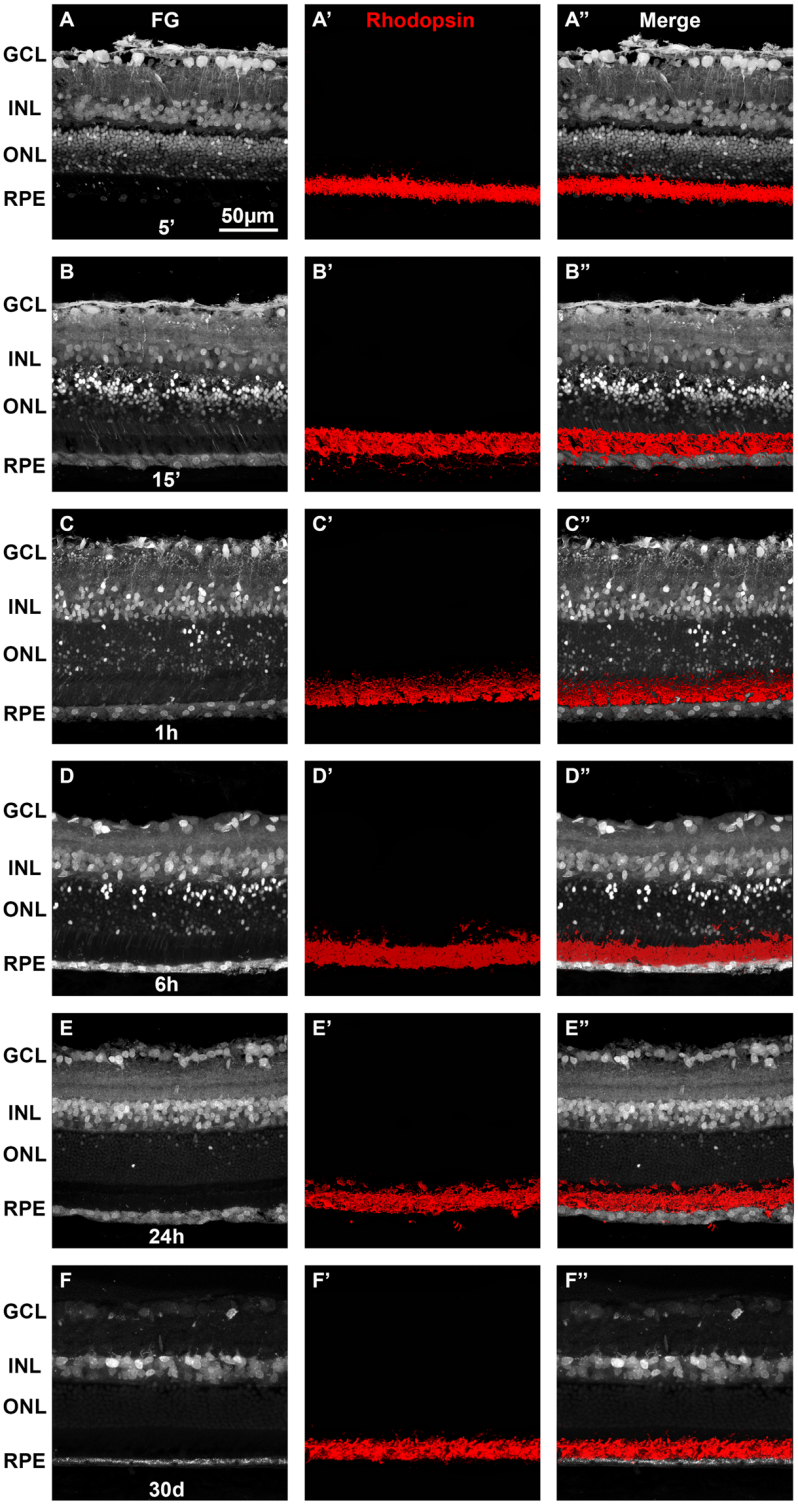
Time course of retinal labeling by intravitreal injection of Fluorogold. Fluorogold tracing in retinal sections from a P60 SD rat tested at 5 (**A**) and 15 (**B**) minutes, 1 (**C**), 6 (**D**) and 24 hours (**E**) and 30 days (**F**) after intravitreal administration of FG. Immunodetection of rhodopsin (**A’**–**F’**) shows that the rod outer segments are not structurally affected by the tracing. Immunodetection in the same sections as (**A–F)**. (**A”–F’’**) merged images. The RPE is labeled 15 minutes after the administration of FG and remains so until 30 days. GCL: ganglion cell layer. INL: inner nuclear layer. ONL: outer nuclear layer. RPE: retinal pigment epithelium.

Next, we performed a time course of the retinal tracing to assess the best time for analysis after FG administration (Fig. 1). Five minutes after FG injection the tracer had already been taken and almost all retinal layers were labeled except for the photoreceptor outer segments (OS) and the RPE cells (Fig. 1A). The tracer marked intensively somas in the ganglion cell layer (GCL), retinal ganglion cell (RGC) axons, and to a lesser extent, somas in both nuclear layers and processes in the plexiform layers. At fifteen minutes the tracer reached the OS and the RPE (Fig. 1B). At 1 hour, the RGC axons were not labeled anymore (Fig. 1C) but cells in both nuclear layers, the OS and the RPE were clearly traced. At six hours (Fig. 1D) the tracing was similar to 1 hour. At 24 h (Fig. 1E) the labeling of the GCL, inner nuclear layer (INL) and RPE was similar to 1 and 6 hours but the outer nuclear layer (ONL) had fewer traced somas and the OS were no longer labeled. Thirty days after FG administration, few cells in the GCL were traced, while the INL and the RPE remained well labeled.

### Intravitreal administration of FG traces retinal neurons and RPE cells but not glial cells

**Figure 2:**
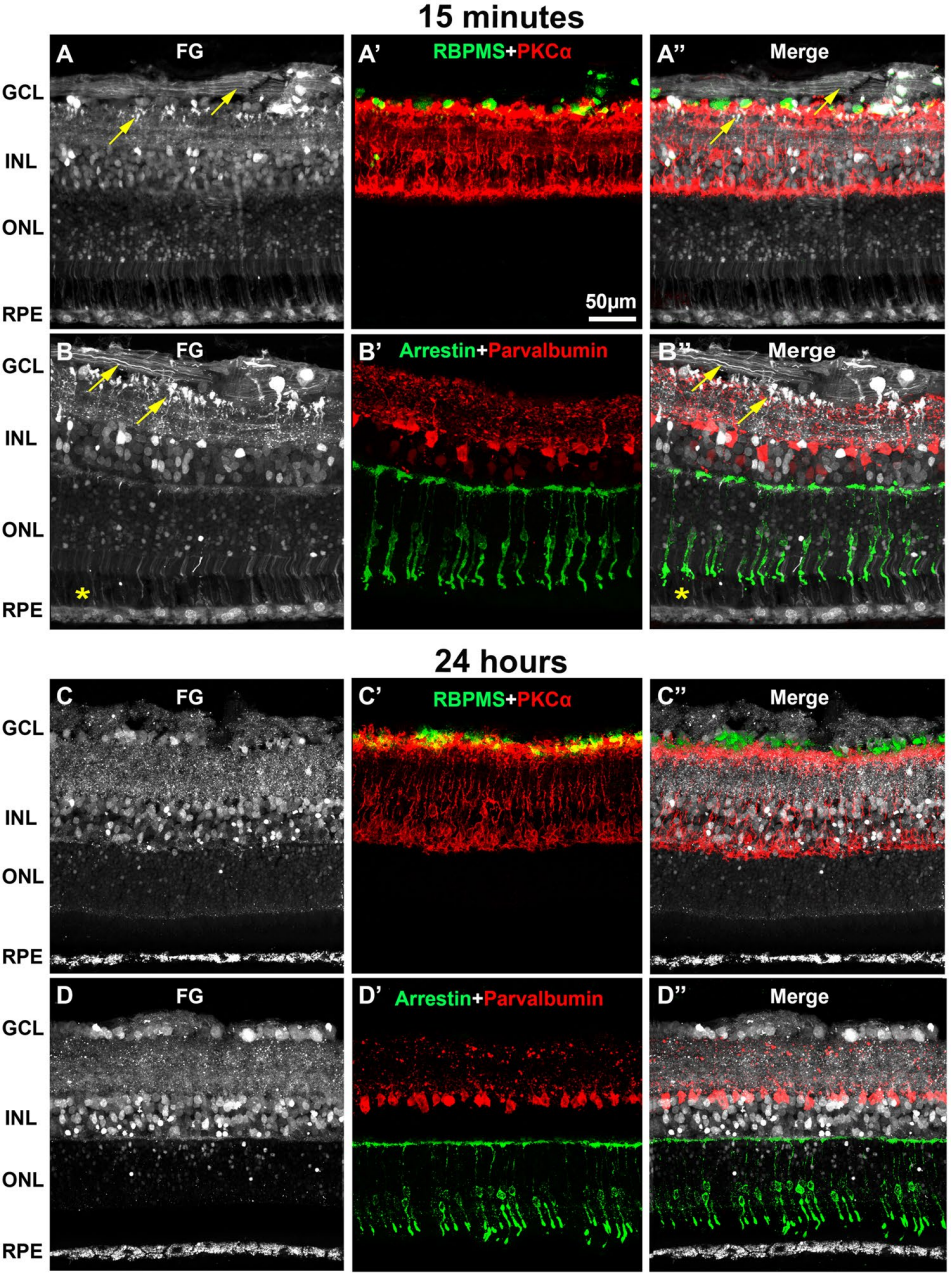
Intravitreally administered fluorogold traces retinal neurons and the retinal pigment epithelium. Representative retinal cross-sections showing fluorogold tracing in young SD rats 15 minutes (**A,B**) or 24 hours (**C,D**) after intravitreal administration. Immunodetection of RBPMS and PKCα (**A’,C’**), arrestin and parvalbumin (**B’,D’**) shows that FG labels RGCs, rod-bipolar cells, cone photoreceptors and amacrine cells, respectively. (**A”–D”)**: merged images. Fifteen minutes after the intravitreal injection, FG tracing is observed in all the retinal layers (**A,B”**), including the axons of RGCs, amacrine cells and rod bipolar cells (yellow arrows) and the outer segments of photoreceptors (yellow asterisks). Twenty-four hours after the injection, FG tracing in RGC axons is fainter and has disappeared from the photoreceptor outer and inner segments. In turn, the RPE is better traced (**C,D”**). GCL: ganglion cell layer. INL: inner nuclear layer. ONL: outer nuclear layer. RPE: retinal pigment epithelium.

**Figure 3:**
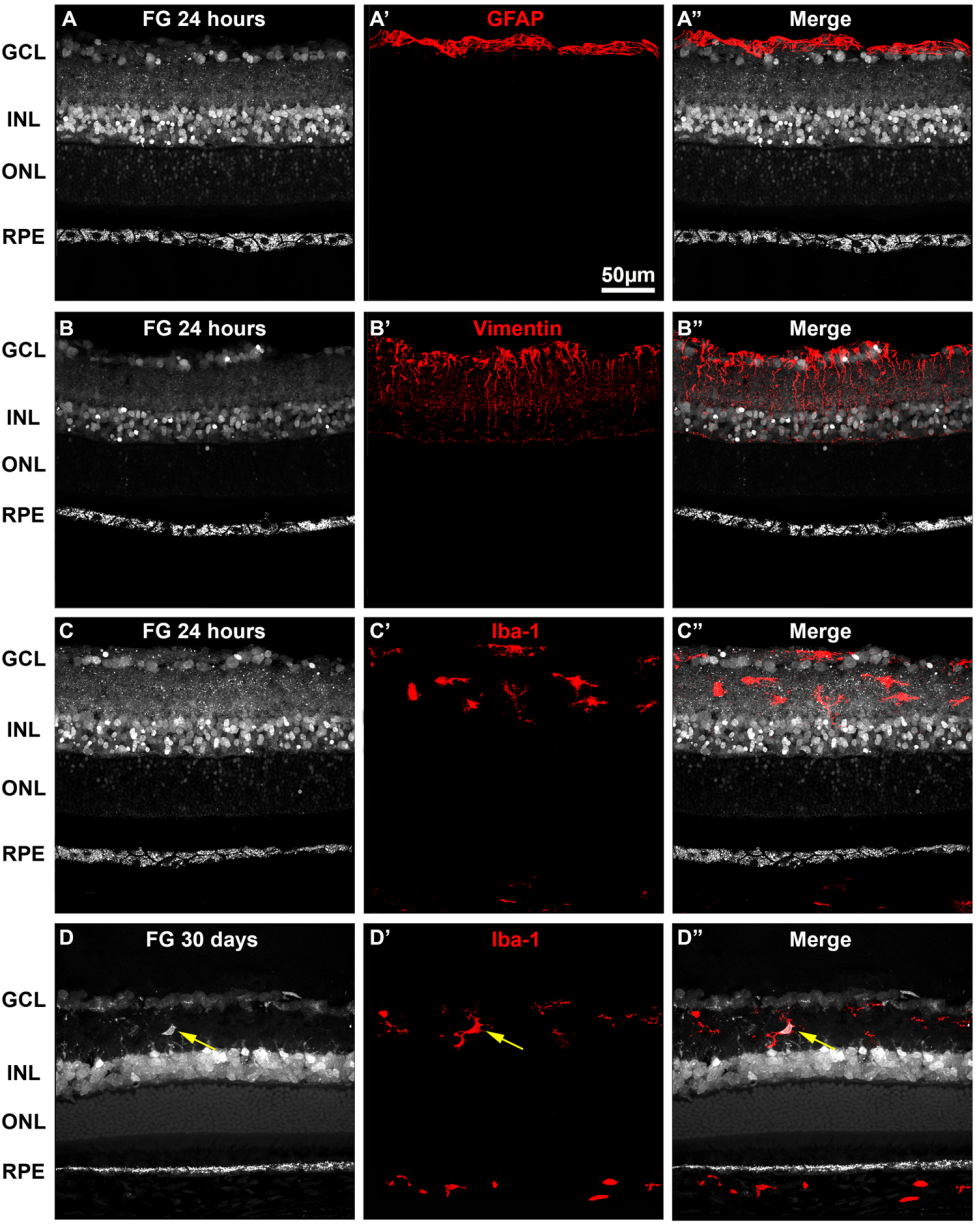
Intravitreally administered fluorogold does not trace the retinal glia. Representative retinal cross-sections showing FG tracing 24 hours after intravitreal administration in young SD rats (**A–C**). Immunodetection of GFAP (**A’**), vimentin (**B’**) or Iba-1 (**C’**) shows that neither astrocytes nor Müller cells or microglial cells, respectively are traced 24 hours after FG-administration. (**A”–C”**) merged images. Thirty days after FG-administration, some FG ^+^ microglial cells are observed (yellow arrows in **D’-D”**). GCL: ganglion cell layer. INL: inner nuclear layer. ONL: outer nuclear layer. RPE: retinal pigment epithelium.

To assess which cells were traced and when, we immunodetected neuronal and glial populations in retinas traced for 15 minutes, 24 hours or 30 days (Figs. 1–3).

Immunodetection of RBPMS (RGCs), PKCα (rod-bipolar cells), parvalbumin (amacrine cells), arrestin (cone photoreceptors) (Fig. 2) and rhodopsin (rods photoreceptors, Fig. 1) showed that 15 minutes after administration, FG had already filled the somas of RGCs, rod-bipolar cells, amacrine cells and cones (Figs. 1B-B”, 2A-B”). In the GCL, a small number of traced somas were not double labeled with RBPMS; these probably correspond to displaced amacrine cells (Fig. 2A-B”). The neuropil in both plexiform layers was also nicely labeled, and the axonal terminals of some amacrines and bipolar cells as well as the inner and OS of some photoreceptors were clearly delineated (Figs. 1B-B”, 2A-B”, yellow arrows). The tracer finally reaches the RPE which at this time point, is faintly labeled (Figs. 1B-B”, 2A-B”).

As shown in the time-course experiment, twenty-four hours after FG administration the pattern of labeling was quite different from 15 minutes of tracing: the axons of the RGCs and the axonal terminals of the bipolar and amacrine cells were scarcely traced, while the somas in the RGC layer and the inner nuclear layer were now more strongly labeled (Figs. 1E-E”, 2C-D”). The processes of the inner plexiform layer remained labeled, but in a punctuated manner that did not delineate any them. Although a few photoreceptor somas were still traced, their inner and outer segments were devoid of FG, while the RPE cells accumulated more FG-filled vesicles (Figs. 1E-E”, 2C-D”). Because at this time point the RPE was better traced, we established 24 hours as the standard protocol. Immunodetection of GFAP (astrocytes and activated Müller cells), Vimentin (Müller cells) and Iba-1 (microglial cells) showed that none of these antibodies co-localized with the tracer, and thus that glial cells were not labeled 24 hours after application of the tracer (Fig. 3A–C”). Importantly, Müller cells do not express GFAP and microglial cells do not show morphological symptoms of activation indicating that, at this time point, there is not a gliotic response to the tracer. However, in the retinas exposed to the tracer for 30 days, some FG^+^ microglial cells are observed showing that when the tracer is present for a long time in the retina, microglial cells have either pruned some labeled synapses and/or phagocytosed a dead and traced neuron, thus becoming transcellularly labeled (Fig. 3D-D”).

All these data suggest that FG is likely uptaken by RGC axons and displaced amacrine cells, and then is transported retrogradelly through the retinal neurons reaching the last neurons in the circuit, the photoreceptors. We believe that the tracer accumulates in the photoreceptor outer segments which are cleaned by RPE which in turn, stores the tracer since as above mentioned 30 days after administration, the RPE is still traced.

### Long term tracing with FG causes oxidative stress

**Figure 4:**
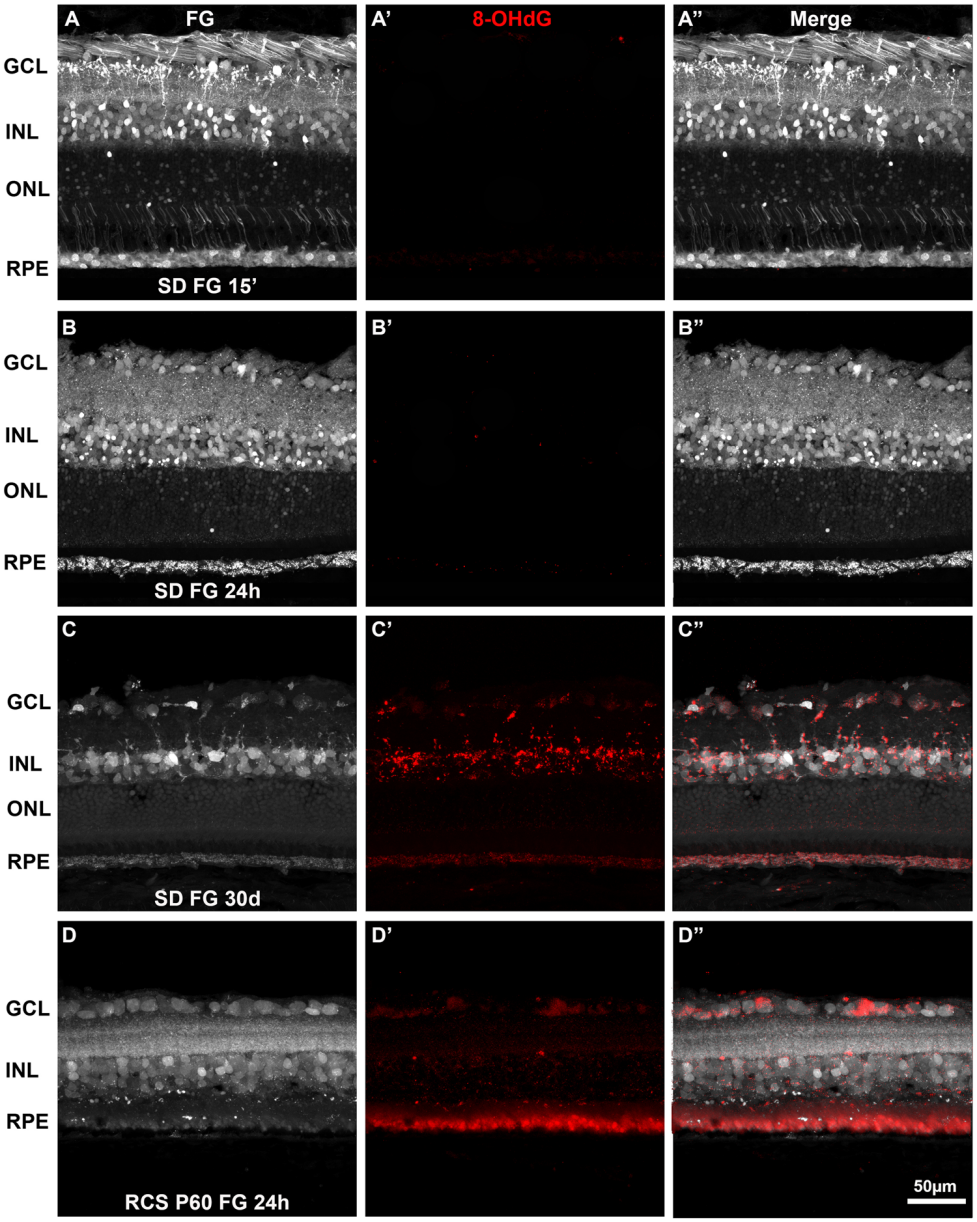
Oxidative stress in the retina after fluorogold tracing. Immunodetection of 8-OHdG in retinal cross-sections analyzed 15 minutes (**A**), 24 hours (**B**) or 30 days (**C**) after intravitreal administration of FG in young SD rats. At 15 minutes (**A’**) or 24 hours (**B’**) there is no 8-OHdG signal. However, at 30 days there is an increase 8-OHdG signal, more evident in the areas with positive FG cells (**C’**). In the positive control, a P60 RCS retinal section, 8-OHdG immunodetection is observed throughout the retina most clearly in the photoreceptor outer segments (**D-D’**). (**A”–D”**) merged images. GCL: ganglion cell layer. INL: inner nuclear layer. ONL: outer nuclear layer. RPE: retinal pigment epithelium.

To study a possible activation of oxidative stress by the continued presence of FG in the retina, retinal sections labeled with FG at 15 minutes, 24 hours or 30 days were immunodetected against 8-OHdG, an oxidative DNA damage marker. Fifteen minutes or 24 hours after FG administration, no 8-OHdG signal was observed in the retina (Fig. 4A,B”). However, a significant increase of 8-OHdG signal was observed throughout the retina when the tracer remains in the retina for 30 days **(**Fig. 4C-C”**)**. This increase was also evident in the positive control (cross sections for a P60 RCS rat; Fig. 4D-D”).

### Aging does not affect the intraretinal tracing with fluorogold

**Figure 5:**
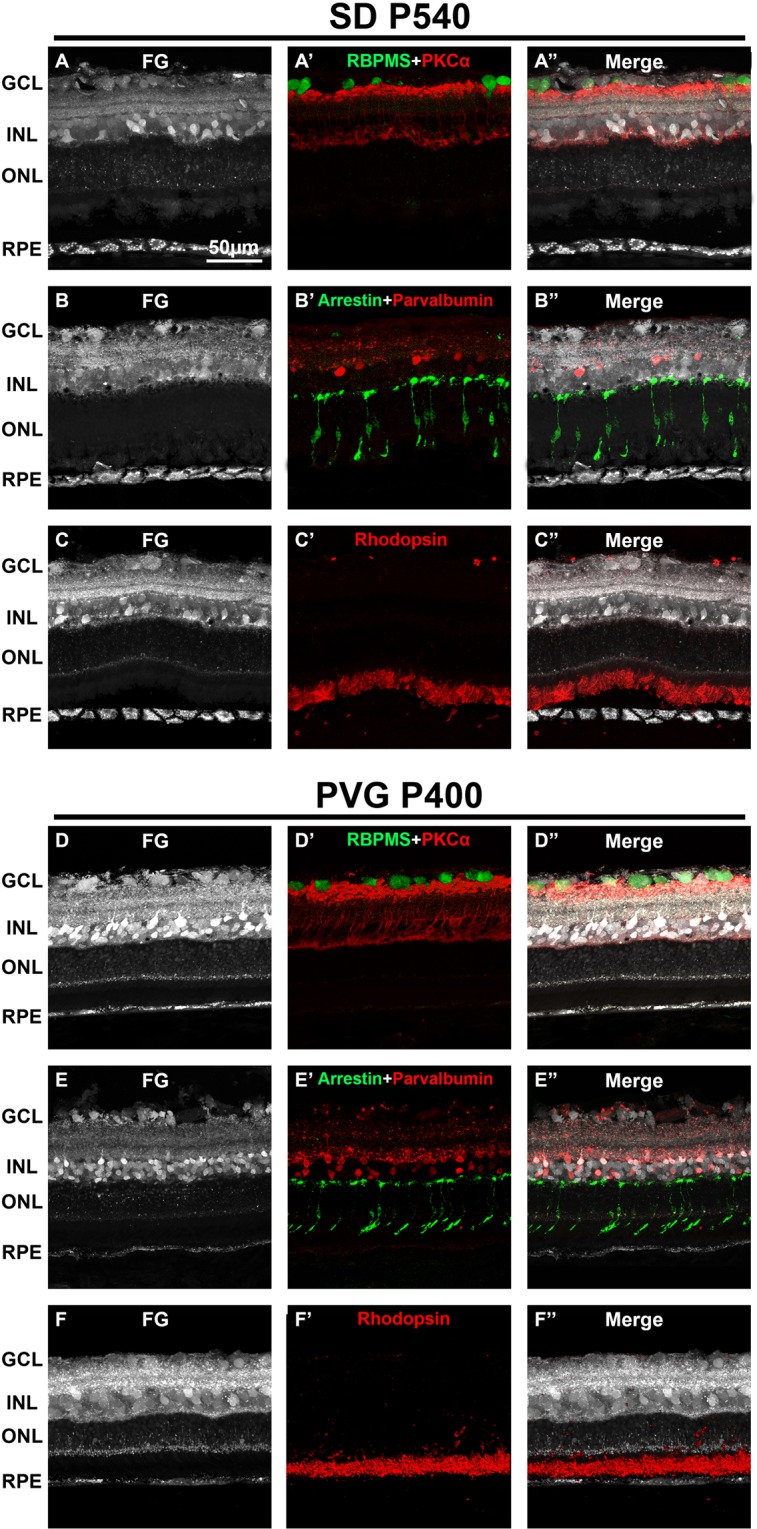
Age does not affect the retinal tracing with fluorogold. Representative retinal cross-sections showing FG tracing in albino P540 SD rats (**A–C**) and in pigmented P400 PVG rats (**D–F**) 24 h after intravitreal administration. Immunodetection of RBPMS and PKCα (**A’,D’**), arrestin and parvalbumin (**B’, E’**) and rhodopsin (**C’-F’**) shows that fluorogold labels the somas RGCs, rod-bipolar cells, cone photoreceptors and amacrine cells, respectively. Rod outer segments are not labeled 24 hours after administration (see Fig. 1). (**A”–F”**) merged images. GCL: ganglion cell layer. INL: inner nuclear layer. ONL: outer nuclear layer. RPE: retinal pigment epithelium. P: postnatal day.

To assess whether aging affected the labeling process, RGCs (RBPMS), rod-bipolar cells (PKCα), amacrine cells (parvalbumin), rods (rhodopsin) and cones photoreceptors (arrestin) were immunodetected in old albino (SD) and pigmented (PVG) control rats. As shown in Fig. 5, the tracing in the aged retinas did not differ from that observed in younger animals, indicating that age does not affect the circuitry and uptake of the tracer (compare Figs. 2 and 5).

### *In vivo* analysis of the retina and RPE in control and dystrophic rats

**Figure 6:**
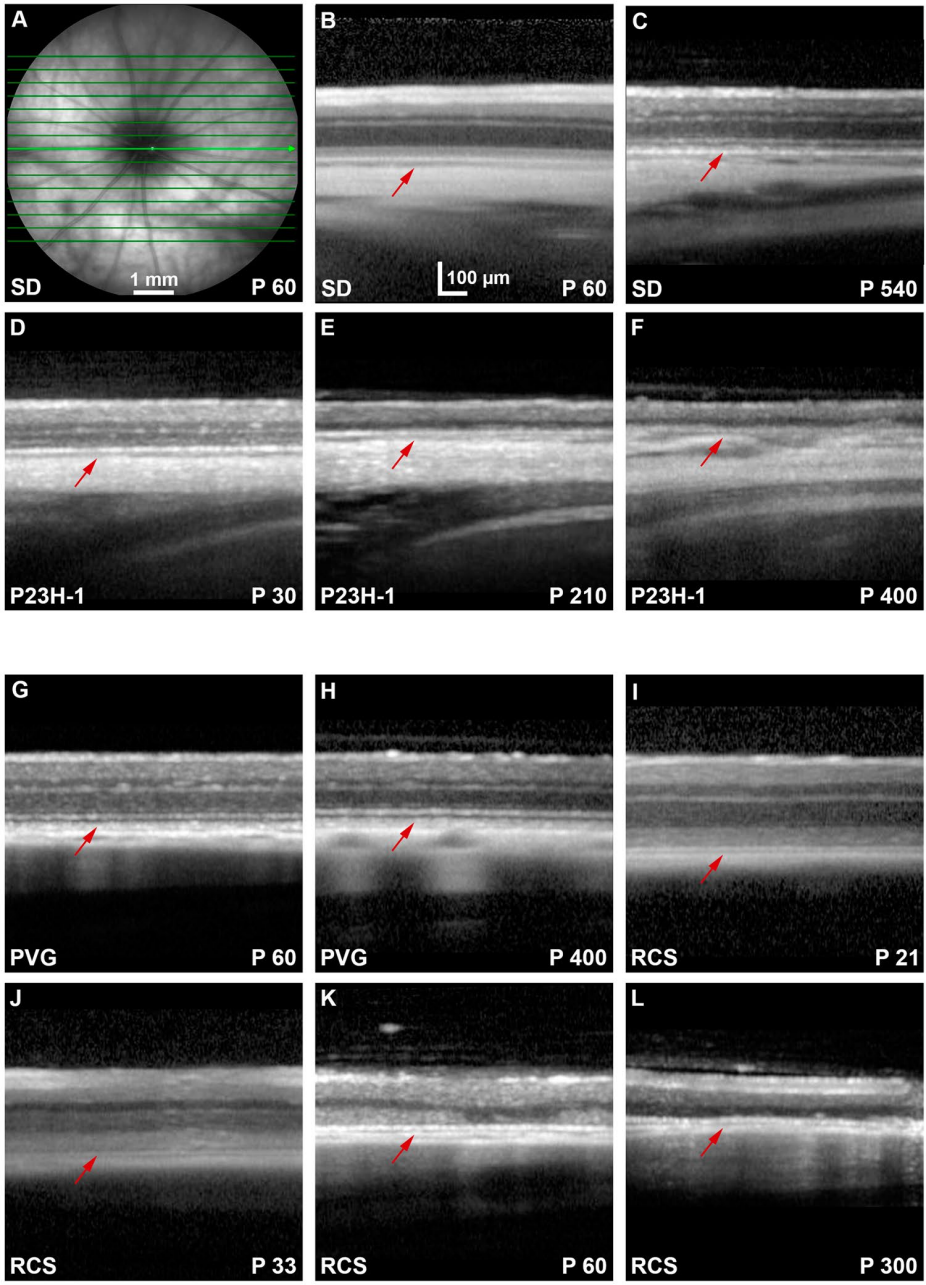
OCT retinal sections in control, P23H-1 and RCS rats. (**A)** Ocular fundus image from a P60 SD rat. The green arrow marks the location of the OCT retinal sections shown in (**B–L)**. (**B–F)** OCT retinal sections encompassing the optic disc acquired from young (**B**) and old (**C**) SD control rats, and 30 (**D**), 210 (**E**) and 400 **(F**) days old P23H-1 rats. (**G–L)** OCT retinal sections encompassing the optic disc acquired from young (**G**) and old (**H**) PVG control rats, and 21 (**I**), 33 (**J**), 60 **(K**) and 300 (**L**) days old RCS rats. In the dystrophic strains is observed that the outer retina diminishes with age. Red arrows point to the position of the RPE. P: postnatal day.

Because the RPE became strongly labeled with FG and we suspected that the passage of FG between the photoreceptors and the RPE cells could be due to the phagocytosis of the outer segments, we injected FG intravitreally in dystrophic rats. Before tracing the retinas of these strains (P23H-1 and RCS) and their respective controls (SD and PVG), animals were analyzed *in vivo* with SD-OCT and FAF. In the SD-OCT sections is observed that in both control strains, the retina thins slightly with age, as previously reported by our group^62^ (Fig. 6B,C,G–H). The P23H-1 rat retina was at the earliest time point examined (P30) thinner than the retinas of control (SD) and RCS rats at this same time point (Fig. 6; compare panels **D** with **B** and **G**) and that the retinas of older control rats (Fig. 6; compare **D** with **C** and **H**). The retinal thinning in P23H-1 rats was due to a decreased thickness of the ONL. This has been observed before by our group and is probably caused by the photoreceptor loss that has already started at this time point in this strain^59^ (Fig. 6D–F). In both dystrophic strains, the retina thinned with increased survival times. In P23H-1 rats, at P210 the ONL was absent, and at P400 just the inner retina remained (Fig. 6E,F). In the RCS rats, at P21 the retina had a normal morphology and was thicker than in P60 control animals as reported^62^. In P60 RCS retinas the thickness of the ONL had decreased compared to P33, and at P300 the ONL had completely disappeared (Fig. 6I–L).

**Figure 7:**
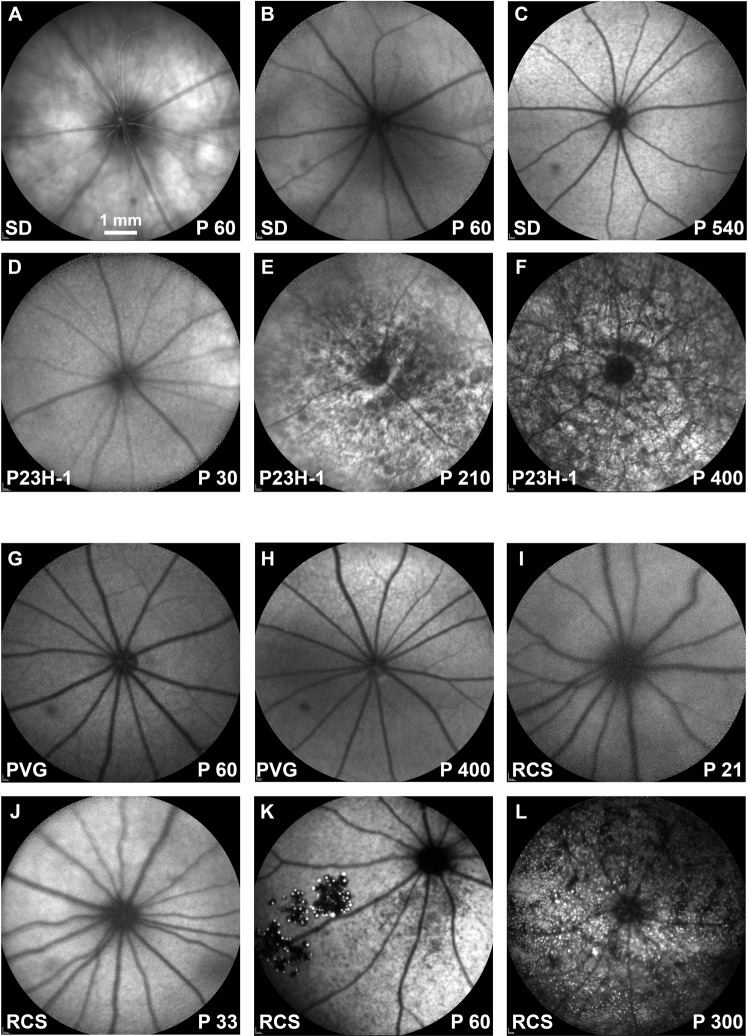
Ocular fundus and BluePeak autofluorescence in control, P23H-1 and RCS rats. (**A)** Representative image from the ocular fundus of a young SD rat. **(B–F**) BluePeak autofluorescence signal acquired from young (**B**) and old (**C**) SD control rats, and 30 (**D**), 210 (**E**) and 400 **(F**) days old P23H-1 rats. (**G-L**) BluePeak autofluorescence signal acquired from young (**G**) and old (**H**) PVG control rats, and 21 (**I**), 33 (**J**), 60 **(K**) and 300 (**L**) days old RCS rats. Fundus autofluorescence represented by autofluorescent dots are seen from P210 onwards in the P23H-1 rat (**E,F**) and at P60 onwards in the RCS rat (**K,L**). P: postnatal day.

FAF examination showed increased RPE autofluorescence in old control animals (Fig. 7B,C,G,H). In the P23H-1 rat, we found normal FAF at P30 (Fig. 7D), but increased dotted FAF at P210 and P400 that may represent lipofuscin accumulations (Fig. 7E,F). In RCS rats, at P21 FAF was similar to control rats (Fig. 7, compare panels G and I). At P33, there was increased diffuse FAF (Fig. 7I,J), at P60 there was dotted FAF mainly in the ventral retinal^23,24,63,64^ and at P300 this dotted pattern occupied all the retina (Fig. 7K,L).

### Intra-retinal tracing in dystrophic retinas

Next, we traced the same rats analyzed *in vivo*. These animals were processed 24 hours after FG tracing, and analyzed in retinal cross sections or RPE flat mounts.

### Cross sections analyses

**Figure 8:**
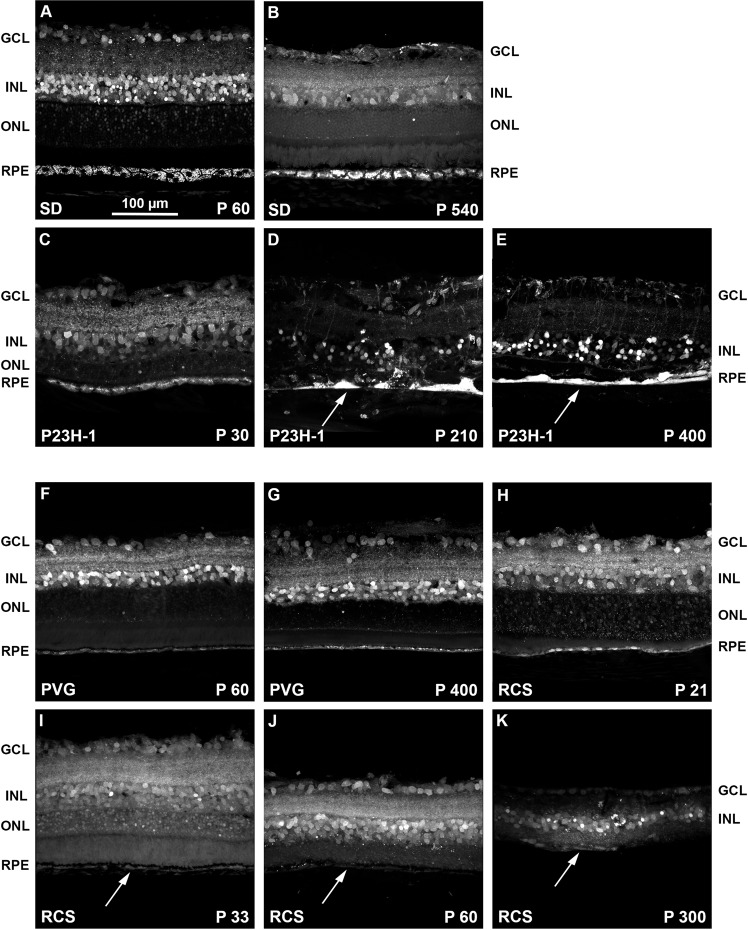
Intraretinal fluorogold tracing shows the retinal remodeling of the dystrophic strains. (**A–E)** Magnifications from retinal cross-sections showing fluorogold tracing in young (**A**) and old (**B**) SD control rats, and 30 (**C**), 210 (**D**) and 400 **(E**) days old P23H-1 rats. (**F–K)** Magnifications from retinal cross-sections showing fluorogold tracing in young (**F**) and old (**G**) PVG control rats, and 21 (**H**), 33 (**I**), 60 **(J**) and 300 (**K**) days old RCS rats. In the P23H-1 retinas, aberrations in the retinal layered structure, and the RPE morphology (white arrows in **D,E**) are observed from P210. The RCS retina degenerates earlier, and at P60 the ONL and the RPE have almost disappeared with only some isolated RPE cells remaining (white arrows in **I-K**). P: postnatal day. GCL: ganglion cell layer. INL: inner nuclear layer. ONL: outer nuclear layer. RPE: retinal pigment epithelium.

Defective FG-labeling was observed in both dystrophic rats compared to their controls (Fig. 8). In the P23H-1 retinas, there were remarkable changes in the RPE with age: at P30, the RPE was similar to control rats, but from P210 the RPE cells were heavily filled with FG and forming a continuum, as if they had fused (Fig. 8C–E) and some FG-labeled RPE cells were observed migrating towards the retina (Fig. 8D) a common finding in this strain^19^. These migrating RPE cells pull down blood vessels, altering the retinal layered structure. Thus, FG tracing is also a good approach to follow the retinal remodeling that takes place following the loss of photoreceptors.

In the RCS strain, the RPE was scarcely labeled at P21, and not labeled at all at older ages (Fig. 8H–K**)**.

**Figure 9:**
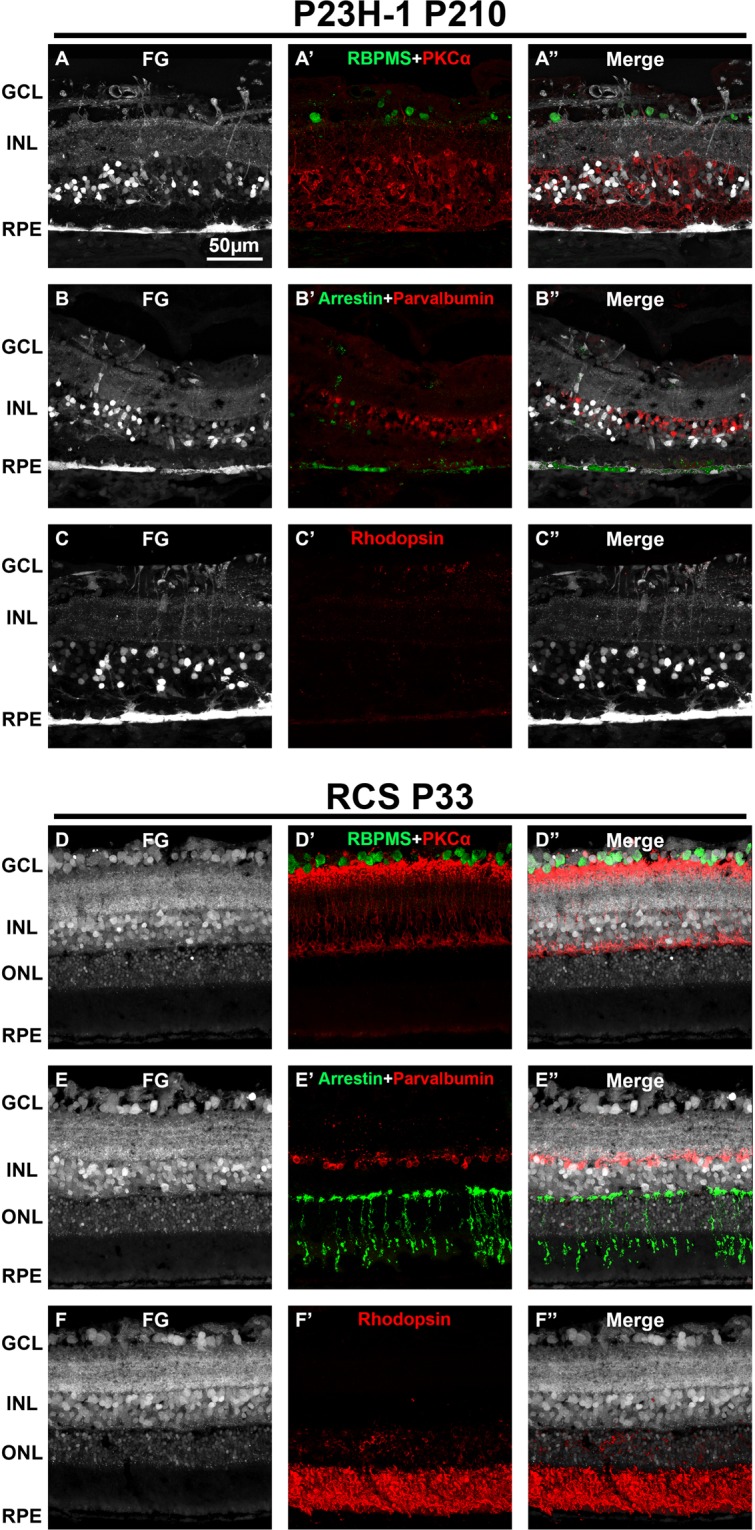
Retinal neurons in traced P23H-1 and RCS rats. Representative retinal cross-sections showing fluorogold tracing in albino P210 P23H-1 rats (**A–C**) and in pigmented P33 RCS rats (**D–F**) 24 h after intravitreal administration of FG. Immunodetection of RBPMS and PKCα (**A’,D’**), arrestin and parvalbumin (**B’, E’**) and rhodopsin (**C’-F’**) shows a clear alteration of the neural circuit in the P23H-1 retina that is more severe in the external retina. However, this remodeling does not prevent the tracer from reaching the RPE (**A-C’**). The neuronal circuit in the RCS is preserved at P33. However, the RPE is not labeled, indicating that it is not functional (**D-F’**). (**A”-F”**) merged images. GCL: ganglion cell layer. INL: inner nuclear layer. ONL: outer nuclear layer. RPE: retinal pigment epithelium. P: postnatal day.

Next, we looked at the retinal circuit in these strains, to assess whether the defective tracing was due to an impaired circuit or not. We analyzed P210 P23H-1 rats and P33 RCS rats, because at P210, the P23H-1 retina is already being remodeled and the RPE accumulates FG, while in P33 RCS rats the retina has not yet being remodeled but the RPE is not labeled (Fig. 8). As shown in Fig. 9A–C”, in P210 P23H-1 rats there was a pronounced neuronal disorganization, more evident in the outer retina. Interestingly, although neither cones nor rods were left at this post-natal time the RPE was labeled with FG showing its altered integrity (Figs. 7C–E and 9A’–C’). In the P33 RCS retinas **(**Fig. 9D–F”) the retinal circuit was still intact, but despite it, the RPE was not labeled with FG, suggesting that in order to accumulate the tracer, the RPE must be functional.

### Flat mount analyses

**Figure 10:**
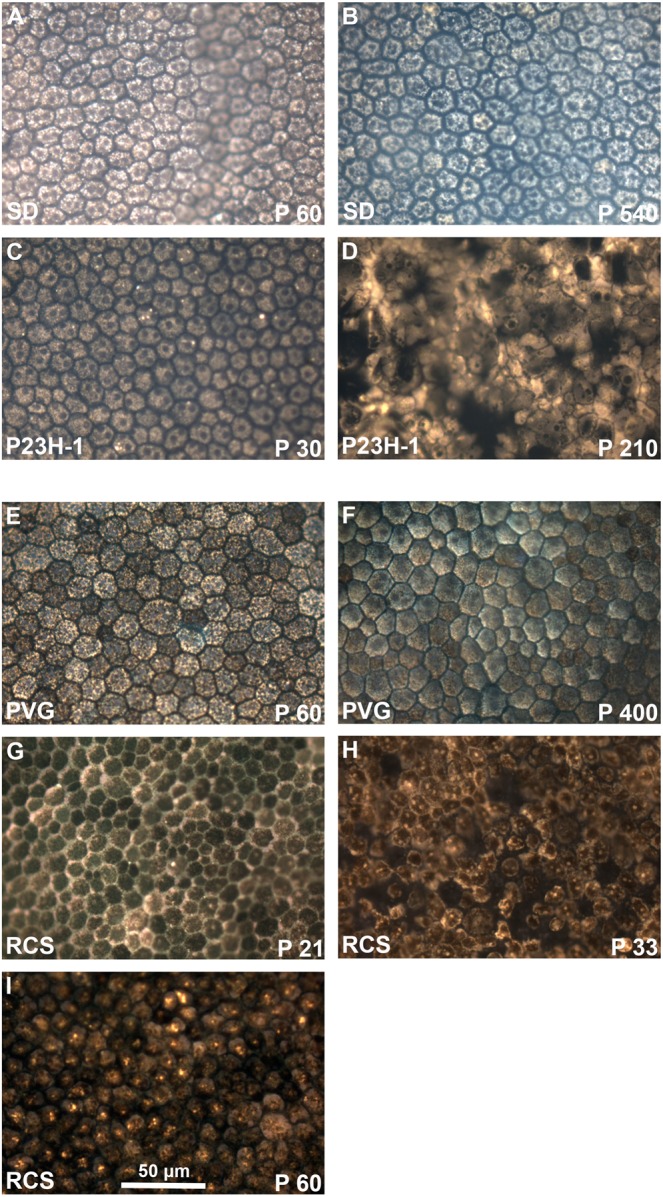
Morphology of the retinal pigment epithelium in flat-mounts: epifluorescence imaging. Magnifications from RPE flat-mounts acquired with an epifluorescence microscope 24 hours after intravitreal administration of fluorogold. (**A–D)** images showing the morphology of traced-RPE cells in young (**A**) and old (**B**) SD control rats, and 30 (**C**) and 210 (**D**) days old P23H-1 rats. (**E–I)** images showing the morphology of traced-RPE cells in young (**E**) and old (**F**) PVG control rats, and 21 (**G**), 33 (**H**) and 60 (**I**) days old RCS rats. In control retinas, FG is observed inside the RPE cells, delineating their hexagonal morphology. At P210 the morphology of P23H-1 RPE cells is greatly disturbed. In the RCS strain, the RPE is unable of phagocytosis and thus FG accumulates outside the RPE cells. At P21 the honeycomb structure of the RPE is still maintained but from P33 onwards this configuration is lost.

**Figure 11:**
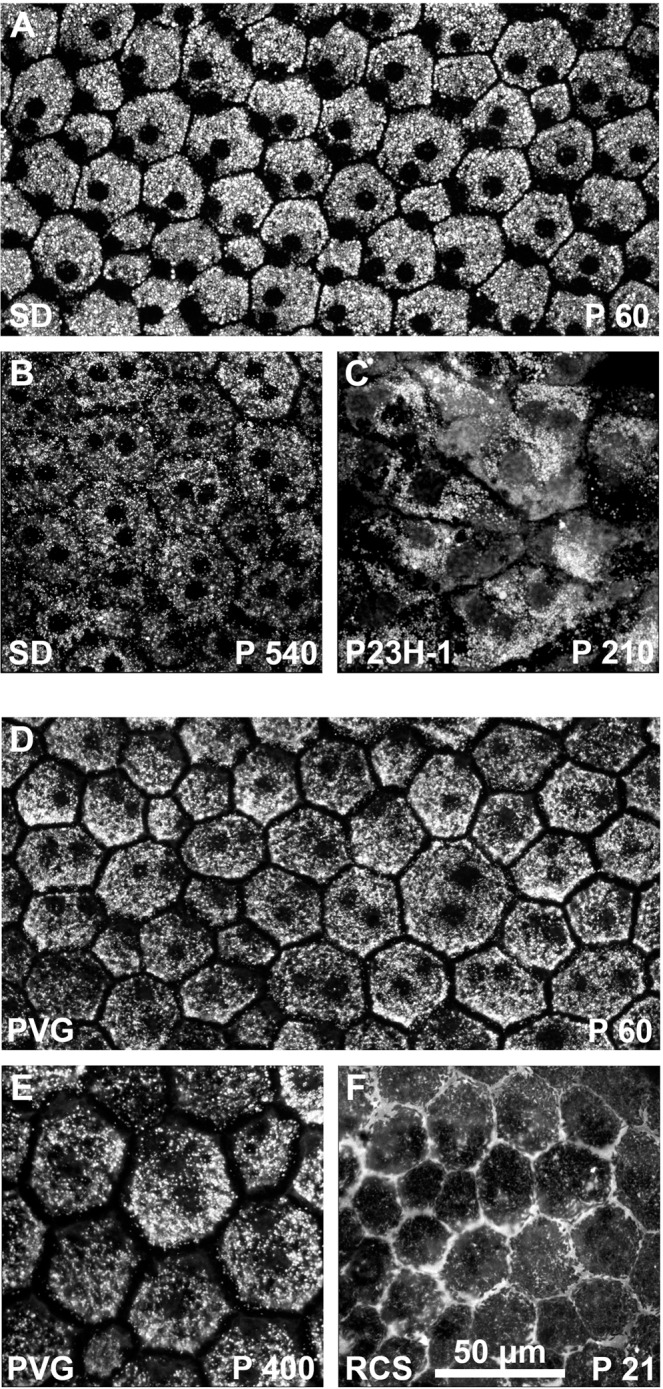
Morphology of the retinal pigment epithelium in flat-mounts: confocal imaging. Magnifications from RPE flat-mounts acquired with a confocal microscope 24 hours after intravitreal administration of fluorogold. (**A–C**) images showing the morphology of traced-RPE cells in young (**A**) and old (**B**) SD control rats, and 210 (**D**) days old P23H-1 rats. (**D–F)** images showing the morphology of traced-RPE cells in young (**D**) and old (**E**) PVG control rats, and 21 (**F**) days old RCS rats. In old SD rats, the RPE is less labeled and there is extracellular FG. In the dystrophic retinas, these images shown the same alterations shown in the previous figure but at higher resolution.

RPE flat mounts were imaged with an epifluorescence and a confocal microscope (Figs. 10 and 11, respectively). In control animals, FG-labeling allowed the visualization of the normal honeycomb distribution of the RPE cells. FG-filled vesicles accumulated in the RPE cytoplasm delineating the nucleus, and no FG was observed between adjacent cells (Figs. 10A,B,E,F, 11A,D). The RPE was better imaged in the albino than the pigmented strain, because in pigmented animals the melanin granules somehow obscured the fluorescence.

When we compared the RPE from young and old control rats, there were no differences at the level of optical microscopy (Fig. 10A,B,E,F**)**. However, when visualized with a confocal microscope we observed that the RPE of old SD rats did not accumulate as much FG as in young rats, and in addition, there was extracellular FG which may indicate that the tight junctions are not working properly (Fig. 11A,B). This was not seen in old PVG rats (Fig. 11D,E), but they were P400 and the albino rats P540.

The RPE cells of P23H-1 rats had a normal morphology at P30. At P210 however, there was loss of the normal hexagonality (pleomorphism and polymegathism) (Figs. 10C,D, 11C) and there were some giant multinucleated cells (compare RPE cells size in panel A and panel C in Fig. 11).

In P21 RCS rats, the RPE showed an inverted labeling: there was FG around the cells (Figs. 10G, 11F), instead of within their somas. At P33 there were some empty spaces that we interpret as spaces devoid of RPE cells. The remaining RPE cells showed pleomorphism and polymegathism and there was also some autofluorescence mostly observed perinuclearly. At P60, the RPE cells show no FG labeling and the degenerative events, polymegathism, pleomorphism and autofluorescence, had further advanced (Fig. 10H,I).

## Discussion

Here we show that the intravitreal administration of FG is a reliable technique to quickly label retinal neurons and RPE cells in rats. This new approach is especially useful to study the RPE not only because the tracer shows the RPE morphology but, most importantly, because it assesses whether the RPE maintains a functional phagocytosis and healthy tight junctions. In addition, this technique also reveals the drastic retinal remodelling that occurs in dystrophic strains^19,58,59^.

Animal models are essential to understand the course of retinal diseases and to test new therapies^59,65,66^. As we show here, OCT and eye fundus autofluorescence imaging in rodents is very useful to perform longitudinal analysis of retinal degeneration^29,30,62^ and RPE metabolic stress, respectively. The longitudinal study of control and dystrophic animals shows *in vivo*, that the retina of control rats thins with age according to previous reports^67^. In the dystrophic strains the OCT sections show, in addition, the pathological thinning caused by the loss of the outer nuclear layer. Both changes are observed as well in the cross sections of the traced retinas^19,58,59^.

The blue peak fluorescence images in elderly control rats show a moderate increase in the signal that indicates accumulation of lipofuscin, and hence the age-related RPE metabolic stress^7,10,29,68^. In the P23H-1 and RCS rats the eye fundus autofluorescence shows that the RPE is severely altered and resembles the hyperrefringent points documented in the macular edema as a sign of atrophy^69–71^.

Intravitreal administration of FG labels the retinal neurons and the RPE in a time and cellular compartment dependent manner: five minutes after the intravitreal injection of FG, the axons, somas and dendrites of RGCs, displaced amacrine cells, and neurons in INL (bipolar and amacrine cells) and ONL (rods and cones) are traced. Fifteen minutes after administration, the tracer also reaches the outer segments of photoreceptors and the RPE. Six hours after the administration, FG labeling accumulates in the neuronal somas in the GCL and INL but has disappeared from the axons. Twenty-four hours after the administration, the labeling of the ONL has decreased, and there are not traced outer segments any longer. However, the RPE is more strongly labeled and this tracing lasts at least up to 30 days after its administration. We chose 24 hours of tracing because the focus of this work was to analyze the RPE. However shorter tracing times may be used to study other retinal layers. For instance, to study RGC axons the tracing should not be longer than 15 minutes.

The accumulation of FG first in axons and then in somas, supports the idea that the tracer travels retrogradely through neurons, a hypothesis that is strengthen by the lack of tracing in glial cells and in the retinal parenchyma. The RPE is in close contact with the photoreceptor’s outer segments, and the tracer may pass from one to another through active transport or RPE phagocytosis. We think that the labeling of RPE is dependent on its phagocytic capacity because RPE of the RCS strain does not accumulate FG in the cytoplasm. This is further supported by the fact that in P33 RCS rats that still have an intact neuronal circuitry, the RPE is not traced thus discarding that the RPE labeling occurs by diffusion and reinforcing the idea that to incorporate the tracer the RPE must be functional.

The mechanism of FG uptake is not clear. FG is lipophilic molecule, and it is believed that crosses cell membranes entering the cell by endocytosis. Once inside the cells it is packed into lysosomes and endosomes by a favourable pH-gradient^54,55,72^. FG is not uptaken by myelinated axons^49^ unless it is dissolved in an organic solvent such as dimethylsulfoxide^52^. Intraretinal axons are not myelinated, and thus as we show here, they are able to incorporate the tracer dissolved in saline. The next question is how the FG is transported from neuron to neuron. To answer it, more experiments should be done to discern between several possible scenarios, such as transsynaptic transport, or release and uptake of FG-filled vesicles, i.e. exosome-mediated transport.

FG tracing is widely used to label RGCs and it does not cause RGC toxicity even when the retinas are analyzed months after the tracing^52^. However, we show here that in retinas analyzed at 30 days after the tracing there is an increased expression of 8-OHdG, a marker of DNA oxidative damage^73–76^. Nevertheless, at 24 hours, which is the established time of tracing for this new method, there is not Müller cell gliosis, microglial activation nor oxidative DNA damage. These data are in concordance with a recent study^77^ showing that the presence of FG in explanted retinas did not induced retinal toxicity, neuronal loss or increase of oxidative stress from 24 hours to 7 days *in vitro*. Thus, intraretinal tracing with FG is a safe method to perform anatomical analysis of the retina and the RPE.

FG tracing beautifully brings out the classical honeycomb pattern of hexagonal RPE cells densely packed along the entire retina^1,7,9,12^. Interestingly, we observed, for the first time, that very old albino rats accumulate less FG indicative of either a slower neuron to neuron transport or of a less phagocytic RPE or both. Furthermore, in these old RPEs there are FG granules between cells, suggesting a lassitude of the tight junctions that, when healthy, make the RPE the sealed barrier between the retina and the choroid. We did not observe these changes in old pigmented control rats, most probably because they were younger than the albino ones.

In the P23H-1 strain, that suffers a mutation in rods but that has a competent RPE we see here that the integrity of the RPE is altered as previously reported with other approaches^29^. These cells show a degenerative morphology and increased size, possibly due to a search for viable photoreceptors.

In the RCS strain the RPE cannot phagocytose leading to photoreceptor degeneration^21,23,24,58–61^. In P21 RCS rats the hexagonal morphology of the RPE cells is still maintained, and the tracer is observed impinging the cytoplasmic membrane but not inside the cell suggestive that the mechanism of FG accumulation in the RPE is via phagocytosis.

In summary, in this work we show for the first time that transneuronal tracing of the retina is a reproducible technique to visualize the retinal neurons in young and old animals, to follow the retinal remodelling in models of retinal dystrophies, and to study the RPE morphology, integrity and functionality.

## Material and Methods

### Animal handling

Animal care and experimental procedures were performed following the Spanish and European Union regulations for the use of animals in research (Council Directive 86/609/EEC) and the ARVO statement for the use of animals in ophthalmic and vision research and were approved by the Ethical and Animal Studies Committee of the University of Murcia (Spain) (numbers: A1320140704 and A13171103).

All rats analyzed in this work were female. Albino Sprague-Dawley (SD) rats and pigmented Piebald Virol Glaxo (PVG) rats were used as controls. The dystrophic strains were the line 1 of the homozygous albino P23H (P23H-1, obtained from M. LaVail (University of California at San Francisco School of Medicine; http://www.ucsfeye.net/mlavailRDratmodels.shtml)^78^ and the homozygous pigmented Royal College of Surgeons (RCS-p1) strains^23,24^. SD, PVG and RCS rats were obtained from the breeding colony of the University of Murcia. Rats were housed and maintained at the animal facilities of the University of Murcia in temperature and light controlled rooms (12 h light/dark cycle) with food and water administered “ad libitum”. Animals were processed at different post-natal days (P) ranging from P21 to P540. Control animals were analyzed at P60 (young) and P400/P540 (old). The ages of the dystrophic strains were chosen based on a previous work describing the course of photoreceptor loss in each one and ranged from P21 to P400^59^. The number of retinas for the characterization of the tracing was 6 per time point. For the study of dystrophic rats, 10 retinas of each strain (including controls) were used (6 for cryostat cross-sections and 4 for flat-mounted RPE).

Intravitreal administration was carried out under general anesthesia administered intraperitoneally by an injection of a mixture of ketamine (60 mg/kg, Ketolar, Parke-Davies, S.L., Barcelona, Spain) and xylazine (10 mg/kg, Rompún, Bayer S.A., Barcelona, Spain). To prevent corneal dryness, Tobrex ointment (Tobrex, Alcon Cusí S.A. Barcelona, Spain) was applied after surgical manipulations.

### Intravitreal administration of fluorogold

Intravitreal injection of 1.5 µl of 3% Fluorogold (FG, Fluorochrome Inc., Engelwood, CO, USA) diluted in saline was performed as previously described^56,79,80^. Briefly, Hamilton micro syringe (30 G; Hamilton 701 N, Esslab, Benfleet, UK) were used to perform the injections through the supratemporal sclera. Before injecting the fluid, we made sure that the needle was correctly positioned. Pilot experiments were done with volumes ranging from 1.5 to 5 µl (Supplementary Data 1).

### Spectral Domain Optical Coherence Tomography (SD-OCT)

Before the acquisition of the images, the pupils of the rats were dilated, and a custom-made contact lens was placed on the cornea to uniform the optics. SD-OCT device (Spectralis; Heidelberg Engineering, Heidelberg, Germany) was used to acquire infrared reflectance imaging of the fundus eye, blue laser (488 nm) autofluorescence imaging, and OCT cross sectional imaging (scan angle 55°). A 78-D double aspheric fundus lens (Volk Optical, Inc., Mentor, OH, USA) was adapted in front of the camera unit to adjust the SD-OCT for the optical qualities of the rat eye. Image acquisition was achieved with a proprietary software package (Eye Explorer, version 3.2.1.0; Heidelberg Engineering, Heidelberg, Germany) and length of the reference pathway was adjusted manually according to manufacturer’s instructions.

### Tissue processing

Rats were euthanized from 5 minutes to 30 days after fluorogold administration by a lethal intraperitoneal injection of pentobarbital (Dolethal Vetoquinol, Especialidades Veterinarias, S.A., Alcobendas, Madrid, Spain) and then they were perfused transcardially with saline and 4% paraformaldehyde in 0.1 M phosphate buffer (pH 7.4). Eyes were enucleated and post fixed for 1 extra hour in 4% paraformaldehyde.

Flat mounted RPE was dissected following a modified method previously described for the retina^81–83^. Briefly, first the retina is pulled from the choroid. This is a crucial step to and must be done carefully. Once the retina is extracted the RPE remains stuck to the choroid. The flat mounted RPE is then mounted side up and covered with anti-fading mounting media.

For cryostat cross sections (15 µm), eye-cups were prepared as previously described^59,84^.

### Immunohistofluorescence

Immunodetection in cross sections was carried out following as described in previous studies from our group^56,59,84^. Primary antibodies were: mouse α-RBPMs (1:250, GeneTex, Gtx118619, CA, USA) to detect RGCs, rabbit α-arrestin (1:1000, AB15282; Chemicon-Millipore Iberica, Madrid, Spain) to detect cones, mouse α-parvalbumin (1:500, SWANT, PV235, Switzerland) to detect amacrine cells, rabbit α-PKCα (1:200, ABCAM, ab48004, UK) to detect rod-bipolar cells, mouse anti-rhodopsin (1:1200, 1D4; Sigma-Aldrich, Madrid, Spain) to detect rhodopsin, goat α-GFAP (1:250; C-19: sc-6170; Santa Cruz Biotechnology, Heidelberg, Germany) to detect both astrocytes and Müller cells, goat α-vimentin (1:100, C-20, sc-7557; Santa Cruz Biotechnology, Heidelberg, Germany) to detect Müller cells and rabbit α-Iba1 antibody (1:500; ab178846; Abcam, Cambridge, UK) to detect microglial cells. For the study of oxidative stress, retinal sections were immunostained against mouse α-8-hydroxy-2’-deoxyguanosine (8-OHdG; 1:1000, sc-66036; Santa Cruz Biotechnology, Heidelberg, Germany), a marker for oxidatively damaged proteins and DNA^75^.

Secondary antibodies were: donkey α-mouse Alexa Fluor 488; donkey α-rabbit Alexa Fluor 594; donkey α-rabbit Alexa Fluor 488; donkey α-goat Alexa Fluor 594 and donkey α-mouse Alexa Fluor 594 (Molecular Probes, ThermoFisher, Madrid, Spain). All were used at 1:500 dilution.

### Image analysis

Retinal cross sections and flat mounted RPE were examined and photographed using an epifluorescence microscope (20X or 40X, Axioscop 2 Plus; Zeiss Mikroskopie, Jena, Germany) or with a confocal microscope Leica SP8 (20X, 40X or 63X, Leica Microsytems, Wetzlar, Germany). A 405 nm continuous wave was used to capture confocal FG images, a 488 nm multiline argon-ion argon laser for the confocal green images and a 561 nm yellow DPSS laser for the confocal red images. Of note, for comparative analyses, 8-OHdG were acquired with the same settings. When required, images were further processed using a graphics editing program (Adobe Photoshop CS 8.0.1; Adobe Systems, Inc., San Jose, CA).

## Supplementary information

Supplementary information.
