# Supplementary material for: Tracing the retina to analyze the integrity and phagocytic capacity of the retinal pigment epithelium
Source: Sci Rep. 2020 Apr 29;10:7273. doi: 10.1038/s41598-020-64131-z (PMC7190639; doi:10.1038/s41598-020-64131-z)
Supplement: Supplementary file 1 — Supplementary information. [file 41598_2020_64131_MOESM1_ESM.pdf]

Tracing the retina to analyze the integrity and phagocytic capacity of the retinal pigment epithelium

Francisco J. Valiente-Soriano[*1], Manuel Salinas-Navarro[1], Johnny Di Pierdomenico[1], Diego García-Ayuso[1], Fernando Lucas-Ruiz[1], Isabel Pinilla[2], Nicolás Cuenca[3], Manuel Vidal-Sanz[1], María Paz Villegas-Pérez[1], Marta Agudo-Barriuso[*1]

[1]Departamento de Oftalmología, Facultad de Medicina, Universidad de Murcia and Instituto Murciano de Investigación Biosanitaria-Virgen de la Arrixaca (IMIB-Arrixaca) Murcia, Spain.

[2]Instituto de Investigación Sanitaria Aragón, Aragon Health Sciences Institute, Lozano Blesa University Hospital Zaragoza, Spain.

[3]Departamento de Fisiología, Genética y Microbiología, Universidad de Alicante Alicante, Spain.

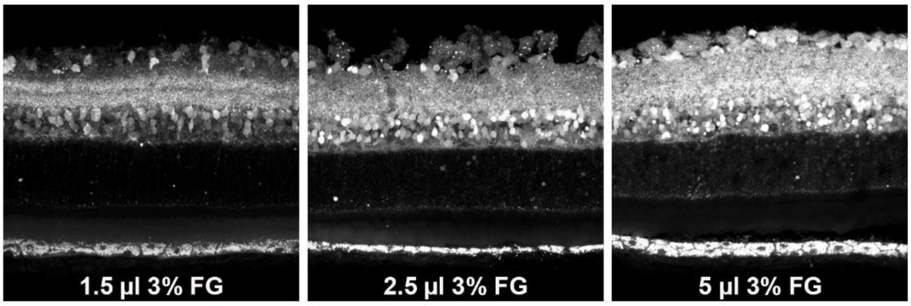

Supplementary Figure: Pilot experiment to adjust the amount of tracer administered intravitreally.

Injections of 1.5, 2.5 or 5 µl FG 3% were tested in cross-sections 24 hours after administration. The RPE was labeled with all volumnes but the best visualization was obtained with 1.5 µl.
